# Supplementary material for: Heat adaptation of phage T7 under an extended genetic code
Source: Virus Evol. 2021 Dec 1;7(2):veab100. doi: 10.1093/ve/veab100 (PMC8923235; doi:10.1093/ve/veab100)
Supplement: veab100_Supp [file veab100_supp.zip › Sequencing_Analysis.zip!/Sequencing_Analysis/S2/evidence/JC_SIDE_2_77.html]

BRESEQ :: Evidence


| New junction evidence | | | | | | | | | | | |
| --- | --- | --- | --- | --- | --- | --- | --- | --- | --- | --- | --- |
|  | | seq id | position | reads (cov) | reads (cov) | score | skew | freq | annotation | gene | product |
| \* | ? | NC\_001604.1 | = 36882 | 5203 (0.980) | 883 (0.170) | 243/582 | 5.8 | 15.5% | intergenic (–/–) | *–/–* | –/– |
|  | ? | NC\_001604.1 | 37265 = | 4556 (0.890) |  |  |  |  | intergenic (–/–) | *–/–* | –/– |
| Rejected: Coverage evenness skew score above cutoff. | | | | | | | | | | | |
| Rejected: Frequency below/above cutoff threshold. | | | | | | | | | | | |

Only 100 of 4878 total aligned reads displayed.  

`CTATTCGGGTTAGGATGGCATCTAGGGTCAGACTCAATGGACGCTAAATGGAAACAGGAGGTACACAATGAGTACGTTAAGAGAGTTGAGGCTGCGAAGAGCACTCAAAGAGCAATCGATGCGGTATCTGCTAAGTATCAAGAAGACCTTGCCGCGCTGGAAGGGAGCACTGATAGGATTATTTCTGATTTGCGTAGCGACAATAAGCGGTTGCGCGTCAGAGTCAAAACTACCGGAACCTCCGATGGTCAGTGTGGATTCGAGCCTGATGGTCGAGCCGAACTTGACGACCGAGATGCTAAACGTATTCTCGCAGTGACCCAGAAGGGTGACGCATGGATTCGTGCGTTACAGGATACTATTCGTGAACTGCAACGTAAGTAGGAAATCAAGTAAGGAGGCAATGTGTCTACTCAATCCAATCGTAATGCGCTCGTAGTGGCGCAACTGAAAGGAGACTTCGTGGCGTTCCTATTCGTCTTATGGAAGGCGCTAAACCTACCGGTGCCCACTAAGTGTCAGATTGACATGGCTAAGGTGCTGGCGAATGGAGACAACAAGAAGTTCATCTTACAGGCTTTCCGTGGTATCGGTAAGTCGT  >  NC_001604.1/36965‑37565`  

                                                                                                                                                                                                                                                                                                            `|`                                                                                                                                                                                                                                                                                                              
       `ggTTAGGATGGCATCTAGGGTCAGACTCAATGGACGCTAAATGGAAACAGGAGGTACACAATGAGTACGTTAAGAGAGTTGAGGCTGCGAAGAGCACTCAAAGAGCAATCGATGCGGTATCTGCTAAGTATCAAGAAGACCTTGCCGCGCTGGAAGGGAGCACTGATAGGATTATTTCTGATTTGCGTAGCGACAATAAGCGGTTGCGCGTCAGAGTCAAAACTACCGGAACCTCCGATGGTCAGTGTGGATTCGAGCCTGATGGTCGAGCCGAACTTGACGACCGAGATGCTAAACGTAt                                                                                                                                                                                                                                                                                                       >  1:144593/1‑301 (MQ=255)`  
                         `ggTCAGAGTCAATGGACGCTAAATGGAAACAGGAGGTACACAATGAGTACGTTAAGAGAGTAGAGGCTGCGAAGAGCACTCAAAGAGCAATCGATGCGGTATCTGCTAAGTATCAAGAAGACCTTGCCGCGCTGGAAGGGAGCACTGATAGGATTATTTCTGATTTGCGTAGCTACAATAAGCGGTTGCGCGTCAGAGTCAAAACTACCGGAACCTCCGATGGTCAGTGTGGATTCGAGCCTGATGGTCGAGCCGAACTTGACGACCGAGATGCTAAACGTATTCTCGCAGTGACCCAGaa                                                                                                                                                                                                                                                                                     <  1:204019/301‑1 (MQ=255)`  
                          `gTCAGACTCAATGGACGCTAAATGGAATCAGGAGGTACACAATGAGTACGTTAAGAGAGTTGAGGCTGCGAAGAGCACTCAAAGAGCAATCGATGCGGTATCTGCTAAGTATCAAGAAGACCTTGCCGCGCTGGAAGGGAGCACTGATAGGATTATTTCTGATTTGCGTAGCGACAATAAGCGGTTGCGCGTCAGAGTCAAAACTACCGGAACCTCCGATGGTCAGTGTGGATTCGAGCCTGATGGTCGAGCCGAACTTGACGACCGAGGTGCTAAAcctgtctcttatacacatctccga                                                                                                                                                                                                                                                                                    >  1:106784/1‑278 (MQ=255)`  
                          `gTCAGACTCAATGGACGCTAAATGGAAACAGGAGGTACACAATGAGTACGTTAAGAGAGTTGAGGCTGCGAAGAGCACTCAAAGAGCAATCGATGCGGTATCTGCTAAGTATCAAGAAGACCTTGCCGCGCTGGAAGGGAGCACTGATAGGATTATTTCTGATTTGCGTAGCGACAATAAGCGGTTGCGCGTCAGAGTCAAAACTACCGGAACCTCCGATGGTCAGTGTGGATTCGAGCCTGATGGTCGAGCCGAACTTGACGACCGAGATGCTAAACGTATTCTCGCAGTGACCCAGAAg                                                                                                                                                                                                                                                                                    >  1:457973/1‑301 (MQ=255)`  
                              `gACTCAATGGACGCTAAATGGAAACAGGAGGTACACAATGAGTACGTTAAGAGAGTTGAGGCTGCGAAGAGCACTCAAAGAGCAATCGATGCGGTATCTTCTAAGTATCAAGAAGACCTTGCCGCGCTGGCAGGGAGCACTGATAGGATTATCTCTGATTTGCGTAGCGACAATAAGCGGTTGCGCGTCAGAGTCAAAACTACCGGAACCTCCGATGGTCAGTGTGGATTCGATCCTGATGGTCGAGCCGAACTTGACGACCGAGATGCTAAACGTATTCTCGCAGTTACCCCGAAGGGTg                                                                                                                                                                                                                                                                                >  1:365011/1‑301 (MQ=255)`  
                              `gACTCAATGGACGCTAAATGGAAACAGGAGGTACACAATGAGTACGTTAAGAGAGTTGAGGCTGCGAAGAGCACTCAAAGAGCAATCGATGCGGTATCTGCTAAGTATCAAGAAGACCTTGCCGCGCTGGAAGGGAGCACTGATAGGATTATTTCTGATTTGCGTAGCGACAATAAGCGGTTGCGCGTCAGAGTCAAAACTACCGGAACCTCCGATGGTCAGTGTGGATTCGAGCCTGATGGTCGAGCCGAACTTGACGACCGAGATGCTAAACGTACTCTCGCAGTGACCCAGaactgtc                                                                                                                                                                                                                                                                                >  1:346619/1‑296 (MQ=255)`  
                                           `cTAAATGGAAACAGGAGGTACACAATGAGTACGTTAAGAGAGTTGAGGCTGCGAAGAGCACTCAAAGAGCAATCGATGCGGTATCTGCTAAGTATCAAGAAGACCTTGCCGCGCTGGAAGGGAGCACTGATAGGATTATTTCTGATTTGCGTAGCGACAATAAGCGGTTGCGCGTCAGAGTCAAAACTACCGGAACCTCCGATGGTCAGTGTGGATTCGAGCCTGATGGTCGAGCCGAACTTGACGACCGAGATGCTAAACGTATTCTCGCAGTGACCCAGAAgggctgtctcttataccc                                                                                                                                                                                                                                                                   >  1:157754/1‑286 (MQ=255)`  
                                             `aaaTGGAAACAGGAGGTACACAATGAGTACGTTAAGAGAGTTGAGGCTGCGAAGAGCACTCAAAGAGCAATCGATGCGGTATCTGCTAAGTATCAAGAAGACCTTGCCGCGCTGGAAGGGAGCACTGATAGGATTATTTCTGATTTGCGTCGCGCCAATAAGCGGTTGCGCGTCAGAGTCAAAACTACCGGAACCTCCGATGGTCAGTGTGGATTCGAGCCTGATGGTCGAGCCAAACTTGACGACCGAGATGCTAAACGTATTCTCGCAGTGACCCAGCAGGGTGACGCATGGATTCGtt                                                                                                                                                                                                                                                                 >  1:786390/1‑300 (MQ=255)`  
                                             `aaaTGGAAACAGGAGGTACACAATGAGTACGTTAAGAGAGTTGAGGCTGCGAAGAGCACTCAAAGAGCAATCGATGCGGTATCTGCTAAGTATCAAGAAGACCTTGCCGCGCTGGAAGGGAGCACTGATAGGATTATTTCTGATTTGCGTAGCGACAATAAGCGGTTGCGCGTCAGAGTCAAAACTACCGGAACCTCCGATGGTCAGTGTGGATTCGAGCCTGATGGTCGAGCCGAACTTGACGACCGAGATGCTAAACGTATTTTCGCAGTGACCCAGAAGGGTGACGCATGGATTCGTg                                                                                                                                                                                                                                                                 >  1:282409/1‑301 (MQ=255)`  
                                             `aaaTGGAAACAGGAGGTACACAATGAGTACGTTAAGAGAGTTGAGGCTGCGAAGAGCACTCAAAGAGCAATCGATGCGGTATCTGCTAAGTATCAAGAAGACCTTGCCGCGCTGGAAGGGAGCACTGATAGGATTATTTCTGATTTGCGTAGCGACAATAAGCGGTTGCGCGTCAGAGTCAAAACTACCGGAACCTCCGATGGTCAGTGTGGATTCGAGCCTGATGGTCGAGCCGAACTTGACGACCGAGATGCTAAACGTATTCTCGCAGTGACCCAGAAGGGTGACGCATGGATTCGtt                                                                                                                                                                                                                                                                 >  1:768513/1‑300 (MQ=255)`  
                                                 `ggAAACAGGAGGTACACAATGAGTACGTTAAGAGAGTTGAGGCTGCGAAGAGCACTCAAAGAGCAATCGATGCGGTATCTGCTAAGTATCAAGAAGACCTTGCCGCGCTGGAAGGGAGCACTGATAGGATTATTTCTGATTTGCGTAGCGACAATAAGCGGTTGCGCGTCAGAGTCAAAACTACCGGAACCTCCGATGGTCAGTGTGGATTCGAGCCTGATGGTCGAGCCGAACTTGACGACCGAGATGCTAAACGTATTCTCGCAGTGACCCAGAAGGGTGACGCATGGATTCGTGCGtt                                                                                                                                                                                                                                                             >  1:822206/1‑301 (MQ=255)`  
                                                    `aaCAGGAGGTACACAATGAGTACGTTAAGAGAGTTGAGGCTGCGAAGAGCACTCAAAGAGCAATCGATGCGGTATCTGCTAAGTATCAAGAAGACCTTGCCGCGCTGGAAGGGAGCACTGATAGGATTATTTCTGATTTGCGTAGCGACAATAAGCGGTTGCGCGTCAGAGTCAAAACTACCGGAACCTCCGATGGTCAGTGTGGATTCGAGCCTGATGGTCGAGCCGAACTTGACGACCGAGATGCTAAACGTATTCTCGCAGTGACCCAGAAGGGGGACGCAGGGATTCGTGCGTTACa                                                                                                                                                                                                                                                          >  1:808397/1‑301 (MQ=255)`  
                                                     `gtgggctcggagatgtgtataagagacaggagTTGAGGCTGCGAAGAGCACTCAAAGAGCAATCGATGCGGTATCTGCTAAGTATCAAGAAGACCTTGCCGCGCTGGAAGGGAGCACTGATAGGATTATTTCTGATTTGCGTAGCGACAATAAGCGGTTGCGCGTCAGAGTCAAAACTACCGGAACCTCCGATGGTCAGTGTGGATTCGAGCCTGATGGTCGAGCCGAACTTGACGACCGAGATGCTAAACGTATTCTCGCAGTGACCCAGAAGGGTGACGCATGGATTCGTGCGTTACAg                                                                                                                                                                                                                                                         <  1:324635/272‑1 (MQ=255)`  
                                                        `acaggTACACAATGAGTACGTTAAGAGAGTTGAGGCTGCGAAGAGCACTCAAAGAGCAATCGATGCGGTATCTGCTAAGTATCAAGAAGACCTTGCCGCGCTGGAAGGGAGCACTGATAGGATTATTTCTGATTTGCGTAGCGACAATAAGCGGTTGCGCGTCAGAGTCAAAACTACCGGAACCTCCGATGGTCAGTGTGGATTCGAGCCTGATGGTCGAGCCGAACTTGACGACCGAGATGTTAAACGTATTCTCGCAGTGACCCAGAAGGGTGACGCATGGATTCGTGCGTTACAGGAt                                                                                                                                                                                                                                                      <  1:802277/299‑1 (MQ=255)`  
                                                         `gaggTACACAATGAGTACGTTAAGAGAGTTGAGGCTGCGAATAGCACTCAAAGAGCAATCGATGCGGTATCTGCTAAGTATCAAGAAGACCTTGCCGCGCTGGAAGGGAGCACTGATAGGATTATTTCTGATTTGCGTAGCGACAATAAGCGGTTGCGCGTCAGAGTCAAAACTACCGGAACCTCCGATGGTCAGTGTGGATTCGAGCCTGATGGTCGAGCCGAACTTGACGACCGAGATGCTAAACGTATTCTCGCAGTGACCCAGAAGGGTGACGCATgctgtctcttatacacatctc                                                                                                                                                                                                                                                     >  1:381031/1‑281 (MQ=255)`  
                                                          `aagagacagaTGAGTACGTTAAGAGAGTTGAGGCTGCGAAGAGCACTCAAAGAGCAATTGATGCGGTATCTGCTAAGTATCAAGAAGACCTTGCCGCGCTGGAAGGGAGCACTGATAGGATTATTTCTGATTTGCGTAGCGACAATAAGCGGTTGCGCGTCAGAGTCAAAACTACCGGAACCTCCGATGGTCAGTGTGGATTCGAGCCTGATGGTCGAGCCGAACTTGACGACCGAGATGCTAAACGTATTCTCGCAGTGACCCAGAAGGGTGACGCATGGATTCGTGCGTTACAGGATAc                                                                                                                                                                                                                                                    <  1:407255/292‑1 (MQ=255)`  
                                                           `ggTACACAATGAGTACGTTAAGAGAGTTGAGGCTGCGAAGAGCACTCAAAGAGCAATCGATGCGGTATCTGCTAAGTATCAAGAAGACCTTGCCGCGCTGGAAGGTAGCACTGATAGGATTATTTCTGATTTGCGTAGCGACAATAATCGGTTGCGCGTCAGAGTCAAAACTACCGGAACCTCCGATGGTCAGTGTGGATTCGAGCCTGATGGTCGAGCCGAACTTGACGACCGAGATGCTAAACGTATTCTCGCAGTGACCCAGAAGGGTGACGGATGGATTGGTGCGTTACAGGATACt                                                                                                                                                                                                                                                   >  1:678078/1‑301 (MQ=255)`  
                                                           `ggTACACAATGAGTACGTTAAGAGAGTTGAGGCTGCGAAGAGCACTCAAAGAGCAATCGATGCGGTATCTGCTAAGTATCAAGAAGACCTTGCCGCGCTGGAAGGGAGCACTGATAGGATTATTTCTGATTTGCGTAGCGACAATAAGCGGTTGCGCGTCAGAGTCAAAACTACCGGAACCTCCGATGGTCAGTGTGGATTCGAGCCTGATGGTCGAGCCGAACTTGACGACCGAGATGCTAAACGTATTCTTGCAGTGACCCAGAAGGGTGACGCATGGATTCGTGCGTTACAGGATACt                                                                                                                                                                                                                                                   >  1:708469/1‑301 (MQ=255)`  
                                                            `gtgtataagagacaggTTAAGAGAGTTGAGGCTGCGAAGAGCACTCAAAGAGCAGTCGATGCGGTATCTGCTAAGTATCAAGAAGACCTTGCCGCGCTGGAAGGGAGCACTGATAGGATTATTTCTGATTTGCGTAGCGACAATAAGCGGTTGCGCGTCAGAGTCAAAACTACCGGAACCTCCGATGGTCAGTGTGGATTCGAGCCTGATGGTCGAGCCGAACTTGACGACCGAGATGCTAAACGTATTCTCGCAGTGACCCAGAAGGGTGACGCATGGATTCGTGCGTTACAGGATACTa                                                                                                                                                                                                                                                  <  1:297069/286‑1 (MQ=255)`  
                                                            `gTACACAATGAGTACGTTAAGAGAGTTGAGGCTGCGAAGAGCACTCAAAGAGCAATCGATGCGGTATCTGCTAAGTATCAAGAAGACCTTGCCGCGCTGGAAGGGAGCACTGATAGGATTATTTCTGATTTGCGTAGCGACAATAAGCGGTTGCGCGTCAGAGTCAAAACTACCGGAACCTCCGATGGTCAGTGTGGATTCGAGCCTGATGGTCGAGCCGAACTTGACGACCGAGATGCTACACGTATTCTCGCAGTGACCCAGAAGGGTGACGCATGGATTCGTGCGTTACAgctgtctc                                                                                                                                                                                                                                                  >  1:151364/1‑294 (MQ=255)`  
                                                             `tataagagacaggcGTTAAGAGAGTTGAGGCTGCGAAGAGCACTCAAAGAGCAATCGATGCGGTATCTGCTAAGTATCAAGAAGACCTTGCCGCGCTGGAAGGGAGCACTGATAGGATTATTTCTGATTTGCGTAGCGACAATAAGCGGTTGCGCGTCAGAGTCAAAACTACCGGAACCTCCGATGGTCAGTGTGGATTCGAGCCTGATGGTCGAGCCGAACTTGACGACCGAGATGCTAAACGTATTCTCGCAGTGACCCAGAAGGGTGACGCATGGATTCGTGCGTTACAGGATACTAt                                                                                                                                                                                                                                                 <  1:762396/288‑1 (MQ=255)`  
                                                             `gtataagagacagcGTTAAGAGAGTTGAGGCTGCGAAGAGCACTCAAAGAGCAATCGATGCGGTATCTGCTAAGTATCAAGAAGACCTTTCCGCGCTGGAAGGGAGCACTGATAGGATTATTTCTGATTTGCGTAGCGACAATAAGCGGTTGCGCGTCAGAGTCAAAACTACCGGAACCTCCGATGGTCAGTGTGGATTCGAGCCTGATGGTCGAGCCGAACTTGACGACCGAGATGCTAAACGTATTCTCGCAGTGACCCAGAAGGGTGACGCATGGATTCGTGCGTTACAGGATACTAt                                                                                                                                                                                                                                                 <  1:248564/288‑1 (MQ=255)`  
                                                             `aacacAATGAGTACGTTAAGAGAGTTGAGGCTGCGAAGAGCACTCAAAGAGCAATCGATGCGGTATCTGCTAAGTATCAAGAAGACCTTGCCGCGCTGGAAGGGAGCACTGATAGGATTATTTCTGATTTGCGTAGCGACAATAAGCGGTTGCGAGTCAGAGTCAAAACTACCGGAACCTCCGATGGTCAGTGTGGATTCGAGCCTGATGGTCGAGCCGAACTTGACGACCGAGATGCTAAACGTATTCTCGCAGTGACCCAGAAGGGTGACGCATGGATTCGTGCGTTACAGGATACTAt                                                                                                                                                                                                                                                 <  1:498649/300‑1 (MQ=255)`  
                                                             `aaagagacaggTACGTTAAGAGAGTTGAGGCTGCGAAGAGCACTCAAAGAGCAATCGATGCGGTATCTGCTAAGTATCAAGAAGACCTTGCCGCGCTGGAAGGGAGCACTGATAGGATTATTTCTGATTTGCGTAGCGACAATAAGCGGTTGCGCGTCAGAGTCAAAACTACCGGAACCTCCGATGGTCAGTGTGGATTCGAGCCTGATGGTCGAGCCGAACTTGACGACCGAGATGCTAAACGTATTCTCGCAGTGACCCAGAAGGGTGACGCATGGATTCGTGCGTTACAGGATACTAt                                                                                                                                                                                                                                                 <  1:196824/291‑1 (MQ=255)`  
                                                              `acacAATGAGTACGTTAAGATAGTTGAGGCTGCGAAGAGCACTCAAAGAGCAATCGATGCGGTATCTGCTAAGTATCAAGAAGACCTTGCCGCGCTGGAAGGGAGCACTGATAGGATTATTTCTGATTTGCGTAGCGACAATAAGCGGTTGCGCGTCAGAGTCAAAACTACCGGAACCTCCGATGGTCAGTGTGGATTCGAGCCTGATGGTCGAGCCGAACTTGACGACCGAGATGCTAAACGTATTCTCGCAGTGACCCAGAAGGGTGACGCATGGATTCGTGCGTTACAGGATACTAtt                                                                                                                                                                                                                                                <  1:656014/301‑1 (MQ=255)`  
                                                              `aaacaATGAGTACGGTAAGAGAGTTGAGGCTGCGAAGAGAACTAAAAGAGCAATCGATGCGGTATCTGCTAAGTATAAAGAAGACCTTGCCGCGCTGGAAGGGAGCACTGATAGGATTATTTCTGATTTGCGTAGCGACAATAAGCGGTTGCGCGTCAGAGTCAAAACTACCGGAACCTCCGATGGTCAGTGTGGATTCGAGCCTGATGGGCGAGCCGAACTTGACGACCTAGATGCTAAACGTATTCTCGCAGGGACCCAGAAGGGGGACGCATGGATTCGTGCGTTACAGGATACTAtt                                                                                                                                                                                                                                                <  1:373567/299‑1 (MQ=255)`  
                                                                 `caATGAGTACGTTAAGAGAGTTGAGGCTGCGAAGAGCACTCAAAGAGCAATCGATGCGGTATCTGCTAAGTATCAAGAAGACCTTGCCGCGCTGGAAGGGAGCACTGATAGGATTATTTCTGATTTGCGTAGCGACAATAAGCGGTTGCGCGTCAGAGTCAAAACTACCGGAACCTCCGATGGTCAGTGTGGATTCGAGCCTGATGGTCGAGCCGAACTTGACGACCGAGATGCTAAACGTATTCTCGCAGTGACCCAGAAGGGTGACGCATGGATTCGTGCGTTACAGGATACTATTCgg                                                                                                                                                                                                                                             >  1:605283/1‑300 (MQ=255)`  
                                                                 `caATGAGTACGTTAAGAGAGTTGAGGCTGCGAAGAGCACTCAAAGAGCAATCGATGCGGTATCTGCTAAGTATCAAGAAGACCTTGCCGCGCTGGAAGGGACCACTGATAGGATTATTTCTGATTTGCGTAGCGACAATAAGCGGTTGCGCGTCAGAGTCAAAACTACCGGAACCTCCGATGGTCAGTGTGGATTCGAGCCTGATGGTCGAGCCGAACTTGACGACCGAGATGCTAAACGTATTCTCGCAGTGACCCAAAAGGGTGACGCATGGATTCGTGCGTTACAGGATACTATTCGt                                                                                                                                                                                                                                             >  1:513644/1‑301 (MQ=255)`  
                                                                     `gAGTACGTTAAGAGAGTTGAGGCTGCGAAGAGCACTCAAAGAGCAATCGATGCGGTATCTGCTAAGTATCAAGAAGACCTTGCCGCGCTGGAATGGAGCACTGATAGGATTATTTCTGATTTGCGTAGCGACAATAAGCGGTTGCGCGTCAGAGTCAAAACTACCGGAACCTCCGATGGTCAGTGTGGATTCGAGCCTGATGGTCGAGCCGAACTTGACGACCGAGATGCTAAACGTATTCTCGCAGTGACCCAGAAGGGTGACGCATGGATTCGTGCGTTACAGGATACTATTCGTGAAc                                                                                                                                                                                                                                         >  1:414710/1‑301 (MQ=255)`  
                                                                          `cGTTAAGAGAGTTGAGGCTGCGAAGAGCACTCAAAGAGCAATCGATGCGGTATCTGCTAAGTATCATGAAGACCTTGCCGCGCTGGAAGGGAGCACTGATAGGATTATTTCTGATTTGCGTAGCGACAATAAGCGGTTGCGCGTCAGAGTCAAAACTACCGGAACCTCCGATGGTCAGTGTGGATTCGAGCCTGATGGTCGAGCCGAACTTGACGACCGAGATGCTAAACGTATTCTCGCAGTGACCCAGAAGGGTGACGCATGGATTCGTGCGTTACAGGATACTATTCGTGAACTGCaa                                                                                                                                                                                                                                    >  1:579549/1‑301 (MQ=255)`  
                                                                          `cGTTAAGAGAGTTGAGGCTGCGAAGAGCACTCAAAGAGCAATCGATGCGGTATCTGCTAAGTATCAAGAAGACCTTGCCGCGCTGGAAGGGAGCACTGATAGGATTATTTCTGATTTGCGTAGCGACAATAAGCGGTTGCGCGTCAGAGTCAAAACTACCGGAACCTCCGATGGTCAGTGTGGATTCGAGCCTGATGGTCGAGCCGAACTTGACGACCGAGATGCTAAACGTATTCTCGCAGTGACCCAGAAGGGTGACGCATGGATTCGTGCGTTACAGGATACTATTCGTGAACTGCaa                                                                                                                                                                                                                                    >  1:423033/1‑301 (MQ=255)`  
                                                                            `ttAAGAGAGTTGAGGCTGCGAAGAGCACTCAAAGAGCAATCGATGCGGTATCTGCTAAGTATCAAGAAGACCTTGCCGCGCTGGAAGGGAGCACTGATAGGATTATTTCTGATTTGCGTAGCGACAATAAGCGGTTGCGCGTCAGAGTCAAAACTACCGGAACCTCCGATGGTCAGTGTGGATTCGAGCCTGATGGTCGAGCCGAACTTGACGACCGAGATGCTAAACGTATTCTCGCAGTGACCCAGAAGGGTGAAGCATGGATTCGTGCGTTACAGGATACTATTCGtacacctgtctc                                                                                                                                                                                                                                  >  1:481186/1‑290 (MQ=255)`  
                                                                             `taatggagTTGAGGCGGCGAAGAGCACTCAAAGAGCAATCGATGCGGTATCTGCTAAGTATCAAGAAGACCTTGCCGCGCTGGAAGGGAGCACTGATAGGATTATTTCTGATTTGCGTAGCGACAATAAGCGGTTGCGCGTCAGAGTCAAAACTACCGGAACCTCCGATGATCAGTGTGGATTCGAGCCTGATGGTCGAGCCGAACTTGACGACCGAGATGCTAAACGTATTCTCGCAGTGACCCAGAAGGGTGACGCATGGATTCGTGCGTTACAGGATACTATTCGTGAACTGCAACGt                                                                                                                                                                                                                                 <  1:331956/296‑1 (MQ=255)`  
                                                                                  `gagTTGAGGCTGCGAAGAGCACTCAAAGAGCAATCGATGCGGTATCTGCTAAGTATCAAGAAGACCTTGCCGCGCTGGAAGGGAGCACTGATAGGATTATTTCTGATTTGCGTAGCGACAATAAGCGGTTGCGCGTCAGAGTCAAAACTACCGGAACCTCCGATGGTCAGTGTGGATTCGAGCCTGATGGTCGAGCCGAACTTGACGACCGAGATGCTAAACGTATTCTCGCAGTGACCCAGCAGGGTGACGCATGGATTCGTGCGTTACAGGATACTATTCGTGAACTGCAACGTAAgca                                                                                                                                                                                                                            >  1:730824/1‑299 (MQ=255)`  
                                                                                   `agTTGAGGCTGCGAAGAGCACTCAAAGAGCAATCGATGCGGTATCTGCTAAGTATCAAGAAGACCTTGCCGCGCTGGAAGGGAGCACTGATAGGATTATTTCTGATTTGCGTAGCGACAATAAGCGGTTGCGCGTCAGAGTCAAAACTACCGGAACCTCCGATGGTCAGTGTGGATTCGAGCCTGATGGTCGAGCCGAACTTGACGACCGAGATGCTAAACGTATTCTCGCAGTGACCCAGAAGGGTGACGCATGGATTCGTGCGTTACAGGATACTATTCGTGAACTGCAATGtctgtct                                                                                                                                                                                                                           >  1:620096/1‑295 (MQ=255)`  
                                                                                                 `ggggctcggagatgtgtataagagacagaTCTGCTAAGTATCAAGAAGACCTTGCCGCGCTGGAAGGGAGCACTGATAGGATTATTTCTGATTTGCGTAGCGACAATAAGCGGTTGCGCGTCAGAGTCAAAACTACCGGAACCTCCGATGGTCAGTGTGGATTCGAGCCTGATGGTCGAGCCGAACTTGACGACCGAGATGCTAAACGTATTCTCGCAGTGACCCAGAAGGGTGACGCATGGATTCGTGCGTTACAGGATACTATTCGTGAACTGCAACGTAAGCAGGAAATCAAGTAagg                                                                                                                                                                                                             <  1:776567/273‑1 (MQ=255)`  
                                                                                                  `gagCACTCAAAGAGCAATCGATGCGGTATCTGCTAAGTATCAAGAAGACCTTGCCGCGCTGGAAGGGAGCACTGATAGGATTATTTCTGATTTGCGTAGCGACAATAAGCGGTTGCGCGTCAGAGTCAAAACTACCGGAACCTCCGATGGTCAGTGTGGATTCGAGCCTGATGGTCGAGCCGAACTTGACGACCGAGATGCTAAACGTATTCTCGCAGTGACCCAGAAGGGTGACGCATGGATTCGTGCGTTACAGGATACTATTCGTGAACTGCAACGTAAGTAGGAAATCAAGTAagga                                                                                                                                                                                                            >  1:61289/1‑301 (MQ=255)`  
                                                                                                     `cACTCAAAGAGCAATCGATGCGGTATCTGCTAAGTATCAAGAAGACCTTGCCGCGCTGGAAGGGAGCACTGATAGGATTATTTCTGATTTGCGTAGCGACAATAAGCGGTTGCGCGTCAGAGTCAAAACTACCGGAACCTCCGATGGTCAGTGTGGATTCGAGCCTGATGGTCGAGCCGAACTTGACGACCGAGATGCTAAACGTATTCTCGCAGTGACCCAGAAGGGTGACGCATGGATTCGTGCGTTACAGGATACTATTCGTGAACTGCAACGTAAGCAGGAAATCAAGTAAGGAGGc                                                                                                                                                                                                         >  1:189794/1‑301 (MQ=255)`  
                                                                                                         `aaaaGAGCAATCGATGCGGTATCTGCTAAGTATCAAGAAGACCTTGCCGCGCTGGAAGGGAGCACTGATAGGATTATTTCTGATTTGCGTAGCGACAATAAGCGGTTGCGCGTCAGAGTCAAAACTACCGGAACCTCCGATGGTCAGTGTGGATTCGAGCCTGATGGTCGAGCCGAACTTGACGACCGAGATGCTAAACGTATTCTCGCAGTGACCCAGAAGGGTGACGCATGGATTCGTGCGTTACAGGATACTATTCGTGAACTGCAACGTAAGCAGGAAATCAAGTAAGGAGGCAAtg                                                                                                                                                                                                     <  1:310869/300‑1 (MQ=255)`  
                                                                                                         `aaaaGAGCAATCGATGCGGTATCTGATAAGTATCAAGAAGACCTTGCCGCGCTGGAAGGGAGCACTAATAGGATTATTTCAGATTTGCGTAGCAACAATAAGCGGTTGCGCGTCAGAGTCAAAACTACCGGAACCTCCGATGGTCAGTGTGGATTCGAGCCTGATAGTCGAGCCGAACTTGACGACCGAGATGCTAAACGTATTCTCGCAGTGACCCAGAAGGGTGACGCATGGATTCGTGCGTTACAGGATACTATTCGTGAACTGCAACGTAAGCAGGAAATCAAGTAAGGAGGCAAtg                                                                                                                                                                                                     <  1:739995/300‑1 (MQ=255)`  
                                                                                                           `tgtgtataagagacaggTATCTGCTAAGTATCAAGAAGACCTTGCCGCGCTGGAAGGGAGCACTGATAGGATTATTTCTGATTTGCGTAGCGACAATAAGCGGTTGCGCGTCAGAGTCAAAACTACCGGAACCTCCGATGGTCAGTGTGGATTCGAGCCTGATGGTCGAGCCGAACTTGACGACCGAGATGCTAAACGTATTCTCGCAGTGACCCAGAAGGGTGACGCATGGATTCGTGCGTTACAGGATACTATTCGTGAACTGCAACGTAAGCAGGAAATCAAGTAAGGAGGAAAtgtg                                                                                                                                                                                                   <  1:233426/286‑1 (MQ=255)`  
                                                                                                                  `gagatgtgtataagagacaggTATCAAGAAGACCTTGCCGCGCTGGAAGGGAGCACTGATAGGATTATTTCTGATTTGCGTAGCGACAATAAGCGGTTGCGCGTCAGAGTCAAAACTACCGGAACCTCCGATGGTCAGTGTGGATTCGAGCCTGATGGTCGAGCCGAACTTGACGACCGAGATGCTAAACGTATTCTCGCAGTGACCCAGAAGGGTGACGCATGGATTCGTGCGTTACAGGATACTATTCGTGAACTGCAACGTAAGTAGGAAATCAAGTAAGGAGGCAATGTGTCTACTc                                                                                                                                                                                            <  1:303770/281‑1 (MQ=255)`  
                                                                                                                  `aTCGATGCGGTATCTTCTAAGTATCAAGGAGACCTTGCCGCGCTGGAAGGGAGCACTGATAGGATTATTTCTGATTTGCGTAGCGACAAGAAGCGGTTGCGCGTCAGAGTCAAAACTACCGGAACCTCCGATGGTCAGTGTGGATTCGAGCCTGATGGTCGAGCCGAACTTGACGACCGAGATGCTAAACGTATTCTCGCAGTGACCCAGAAGGGTGACGCATGGATTCGTGCGTTACAGGATACTATTCGTGAACTGCAACGTAAGCAGGAAATCAAGTAAGGAGGCAATGTGTCTACTc                                                                                                                                                                                            <  1:435563/301‑1 (MQ=255)`  
                                                                                                                  `aTCGATGCGGTATCTGCTAAGTATCAAGAAGACCTTGCCGCGCTGGAAGGGAGCACTGATAGGATTATTTCTGATTTGCGTAGCGACAATAAGCGGTTGCGCGTCAGAGTCAAAACTACCGGAACCTCCGATGGTCAGTGTGGATTCGAGCCTGATGGTCGAGCCGAACTTGACGACCGAGATGCTAAACGTATTCTCGCAGTGACCCAGAAGGGTGACGCATGGATTCGTGCGTTACAGGATACTATTCGTGAACTGCAACGTAAGTAGGAAATCAAGTAAGGAGGCAATGTGTCTACTc                                                                                                                                                                                            <  1:594727/301‑1 (MQ=255)`  
                                                                                                                  `aTCGATGCGGTATCTGCTAAGTATCAAGAAGACCTTGCCGCGCTGGAAGGGAGCACTGATAGGATTATTTCTGATTTGCGTAGCGACAATAAGCGGTTGCGCGTCAGAGTCAAAACTACCGGAACCTCCGATGGTCAGTGTGGATTCGAGCCTGATGGTCGAGCCGAACTTGACGACCGAGATGCTAAACGTATTCTCGCAGTGACCCAGAAGGGTGACGCATGGATTCGTGCGTTACAGGATACTATTCGTGAACTGCAACGTAAGTAGGAAATCAAGTAAGGAGGCAATGTGTCTACTc                                                                                                                                                                                            <  1:670996/301‑1 (MQ=255)`  
                                                                                                                  `aTCGATGCGGTATCTGCTAAGTATCAAGAAGACCTTGCCGCGCTGGAAGGGAGCACTGATAGGATTATTTCTGATTTGCGTAGCGACAATAAGCGGTTGCGCGTCAGAGTCAAAACTACCGGAACCTCCGATGGTCAGTGTGGATTCGAGCCTGATGGTCGAGCCGAACTTGACGACCGAGATGCTAAACGTATTCTCGCAGTGACCCAGAAGGGTGACGCATGGATTCGTGCGTTACAGGATACTATTCGTGAACTGCAACGTAAGCAGGAAATCAAGTAAGGAGGCAATGTGTCTACTc                                                                                                                                                                                            <  1:539588/301‑1 (MQ=255)`  
                                                                                                                  `aTCGATGCGGCATCTGCTAAGTATCAAGAAGACCTGGCCGCGATGGAAGGGAGCACTGATAGGATTATTTCGTATTTGCGTAGCGACAATAAGCGGTTGCGCGTCAGATTCAAAACTACCGGAACCTCCGATGGTCAGTGTGGATTCTAGCCTGATGGTCGAGCCGAACTTGACGACCGAGATGCTAAACGTATTCTCGCAGTGACCCAGAAGGGTGACGCATGGATTAGTGCGTTACAGGATACTATTCGTGAACTGCAACGTAAGCAGGAAATCAAGTAAGGAGGCAATGTGTCTACTc                                                                                                                                                                                            <  1:57202/301‑1 (MQ=255)`  
                                                                                                                       `tgcgtgatgtGCTAAGGTTCAGGCAGGCCCTGTCGCGCTGAACGTGAGCACTGATAGGCGTATTTCTGACTTGCATAGCGACAGTTAGCGGTTTGCCGTCAGCGTCAAAACTCCCGGAACCTCCGGTAGTCAGTGTGGATTCGAGCCTGCTGGTCGGTCCGAACTGGACAACCGAGATGCAAAACGTATTCTCGCATTGACCCAGAAGGGTGACGCATGGATTCGTGCGTTACAGGATACTATTCGTGGACTGCAACGTAAGCAGGAAATCAAGTAAGGAGGCAATGTGTCTACTcaatcc                                                                                                                                                                                       <  1:701164/292‑1 (MQ=255)`  
                                                                                                                          `ggTATCTGCTAAGTATCAAGAAGACCTTGCCGCGCTGGAAGGGAGCACTGATAGGATTATTTCTGATTTGCGTAGCGACAATAAGCGGTTGCGCGTCAGAGTCAAAACTACCGGAACCTCCGATGGTCAGTGTGGATTCGAGCCTGATGGTCGAGCCGAACTTGACGACCGAGATGCTAAACGTATTCTCGCAGTGACCCAGAAGGGTGACGCATGGATTCGTGCGTTACAGGATACTATTCGTGAACTGCAACGTAAGCAGGAAATCAAGTAAGGAGGCAATGTGTCTACTcaatccaat                                                                                                                                                                                    <  1:723881/301‑1 (MQ=255)`  
                                                                                                                            `tATTTGCTAAGTATCAAGAAGACCTTGCCGCGCTGGAAGGGAGCACTGATAGGATTATTTCTGATTTGCGTAGCGACAATAAGCGGTTGCGCGTCAGAGTCAAAACTACCGGAACCTCCGATGGTCAGTGTGGATTCGAGCCTGATGGTCGAGCCGAACTTGACGACCGAGATGCTAAACGTATTCTCGCAGTGACCCAGAAGGGTGACGCATGGATTCGTGCGTTACAGGATACTATTCGTGAACTGCAACGTAAGTAGGAAATCAAGTAAGGAGGCAATGTGTCTACTCAATCCAATCg                                                                                                                                                                                  <  1:649463/301‑1 (MQ=255)`  
                                                                                                                                 `gCTAAGTATCAAGAAGACCTTGCCGCGCTGGAAGGGAGCACTGATAGGATTATTTCTGATTTGCGTAGCGACAATAAGCGGTTGCGCGTCAGAGTCAAAACTACCGGAACCTCCGATGGTCAGTGTGGATTCGAGCCTGATGGTCGAGCCGAACTTGACGACCGAGATGCTAAACGTATTCTCGCAGTGACCCAGAAGGGTGACGCATGGATTCGTGCGTTACAGGATACTATTCGTGAACTGCAACGTAAGTAGGAAATCAAGTAAGGAGGCAATGTGTCTACTcaatcctgtctcttaa                                                                                                                                                                             >  1:693303/1‑291 (MQ=255)`  
                                                                                                                                 `gCTAAGTATCAAGAAGACCTTGCCGCGCTGGAAGGGAGCACTGATAGGATTATTTCTGATTTGCGTAGCGACAATAAGCGGTTGCGCGTCAGAGTCAAAACTACCGGAACCTCCGATGGTCAGTGTGGATTCGAGCCTGATGGTCGAGCCGAACTTGACGACCGAGATGCTAAACGTATTCTCGCAGTGACCCAGAAGGGTGACGCATGGATTCGTGCGTTACAGGATACTATTCGTGAACTGCAACGTAAGCAGGAAATCAAGTAAGGAGGCAATGTGTCTACTCAATCCAATCGTAATg                                                                                                                                                                             <  1:663594/301‑1 (MQ=255)`  
                                                                                                                                                `gaCCTTGCCGCGCTGGAAGGGAGCACTGATAGGATTATTTCTGATTTGCGTAGCGACAATAAGCGGTTGCGCGTCAGAGTCAAAACTACCGGAACCTCCGATGGTCAGTGTGGATTCGAGCCTGATGGTCGAGCCGAACTTGACGACCGAGATGCTAAACGTATTCTCGCAGTGACCCAGAAGGGTGACGCATGGATTCGTGCGTTACAGGATACTATTCGTGAACTGCAACGTAAGCAGGAAATCAAGTAAGGAGGCAATGTGTCTACTCAATCCAATCGTAATGCGCTCGTAGTGgcgc                                                                                                                                                              <  1:212014/301‑1 (MQ=255)`  
                                                                                                                                                  `ccTTGCCGCGCTGGAAGGGAGCACTGATAGGATTATTTCTGATTTGCGTAGCGACAATAAGCGGTTGCGCGTCAGAGTCAAAACTACCGGAACCTCCGATGGTCAGTGTGGATTCGAGCCTGATGGTCGAGCCGAACTTGACGACCGAGATGCTAAACGTATTCTCGCAGTGACCCAGAAGGGTGACGCATGGATTCGTGCGTTACAGGATACTATTCGTGAACTGCAACGTAAGTAGGAAATCAAGTAAGGAGGCAATGTGTCTACTCAATCCAATCGTAATGCGCTCGTAGTGGCGCaa                                                                                                                                                            <  1:92620/301‑1 (MQ=255)`  
                                                                                                                                                              `ggAAGGGAGCACTGATAGGATTATTTCTGATTTGCGTAGCGACAATAAGCGGTTGCGCGTCAGAGTCAAAACTACCGGAACCTCCGATGGTCAGTGTGGATTCGAGCCTGATGGTCGAGCCGAACTTGACGACCGAGATGCTAAACGTATTCTCGAAGTGACCCAGAAGGGTGACGCATGGATTCGTGCGTTACAGGATACTATTCGTGAACTGCAACGTAAGTAGGAAATCAAGTAAGGAGGCAATGTGTCTACTCAATCCAATCGTAATGCGCTCGTAGTGGCGCAACTGAAAGGAGAc                                                                                                                                                >  1:56588/1‑301 (MQ=255)`  
                                                                                                                                                                     `aGCACTGATAGGATTATTTCTGATTTGCGTAGCGACAATAAGCGGTTGCGCGTCAGAGTCAAAACTACCGGAACCTCCGATGGTCAGTGTGGATTCGAGCCTGATGGTCGAGCCGAACTTGACGACCGAGATGCTAAACGTATTCTCGCAGTGACCCAGAAGGGTGACGCATGGATTCGTGCGTTACAGGATACTATTCGTGAACTGCAACGTAAGTAGGAAATCAAGTAAGGAGGCAATGTGTCTACTCAATCCAATCGTAATGCGCTCGTAGTGGCGCAACTGAAAGGAGACTTCGTgg                                                                                                                                         <  1:271807/301‑1 (MQ=255)`  
                                                                                                                                                                         `cTGATAGGATTATTTCTGATTTGAGTAGCGACAATAAGCGGTTGCGCGTCAGAGTCAAAACTACCGGAACCTCCGATGGTCAGTGTGGATTCGAGCCTGATGGTCGAGCCGAACTTGACGACCGAGATGCTAAACGTATTCTCGCAGTGACCCAGAAGGGTGACGCATGGATTCGTGCGTTACAGGATACTATTCGTGAACTGCAACGTAAGTAGGAAATCAAGTAAGGAGGCAATGTGTCTACTCAATCCAATCGTAATGCGCTCGTAGTGGCGCAACTGAAAGGAGACTTCGTGGCGtt                                                                                                                                     >  1:129041/1‑301 (MQ=255)`  
                                                                                                                                                                               `ggATTATTTCTGATTTGCGTAGCGACAATAAGCGGTTGCGCGTCAGAGTCAAAACTACTGGAACTTCCGATGGTCAGTGTGGATTCGAGCCTGATGGTCGAGCCGCACTTGACGACAGAGATGCTATACGTATTCTCGCAGTGACCCAGAAGGGTGACGCATGGTTTCGTGCGTTACAGGATACTATTCGTGAACTGCAACGTAAGCAGGAAATCAAGTAAGTAGGCAATGTGTCTACTCAATCCCATCGTAATGCGTCCGTATTGGTGGACCTGAAAGGCGACTTCGTGTCGTTCCTAtt                                                                                                                               >  1:359113/1‑301 (MQ=255)`  
                                                                                                                                                                                `gATTATTTCTGATTTGCGTAGCGACAATAAGCGGTTGCGCGTCAGAGTCAAAACTACCGGAACCTCCGATGGTCAGTGTGGATTCGAGCCTGATGGTCGAGCCGAACTTGACGACCGAGATGCTAAACGTATTCTCGCAGTGACCCAGAAGGGTGACGCATGGATTCGTGCGTTACAGGATACTATTCGTGAACTGCAACGTAAGCAGGAAATCAAGTAAGGAGGCAATGTGTCTACTCAATCCAATCGTAATGCGCTCGTAGTGGCGCAACTGAAAGGAGACTTCGTGGCGTTCCTATTc                                                                                                                              >  1:238900/1‑301 (MQ=255)`  
                                                                                                                                                                                   `tattTCTGAGTTGCGTAGCGACAATAAGCGGTTGCGCGTCATAGTCAAAACTACCGGAAACTCGGATGGTCAGTGTGGATTCGAGCCTGATGGTCGAGCCGAACTTGACGACCGAGATGCTAAACGTATTCTCTCAGTGACCCAGAAGGGTGACGCATGGATTCGTGCGTTACAGGATACTATTCGTGAACTGCAACGTAAGCAGGAAATCAAGTAAGGAGGCAATGTGTCTACTCAATCCAATCGTAATGCGCTCGTAGTGGCGAAACTGAAAGGAGACTTCGTGGCGTTCCTATTCGTc                                                                                                                           <  1:351742/301‑1 (MQ=255)`  
                                                                                                                                                                                   `tattGCTGATTTGCGTAGCGACAATAAGCGGTTGCGCGTCAGAGTCAAAACTACCGGAACCTCCGATGGTCAGTGTGGATTCGAGCCTGATGGTCGAGCCGAACTTGACGACCGAGATGCTAAACGTATTCTCGCAGTGACCCAGAAGGGTGACGAATGGATTCGTGCGTTACAGGATACTATTCGTGAACTGCAACGTAAGCAGGAAATCAAGTAAGGAGGAAATGTGTCTACTCAATCCAATCGTAATGCGCTCGTAGTGGCGCAACTGAAAGGAGACTTCGTGGCGTTCCTATTCGTc                                                                                                                           <  1:50710/301‑1 (MQ=255)`  
                                                                                                                                                                                         `tGATTTGCGAAGCGAAAATAAGCGGTTGTGCGTCAGAGTCAAAACTACCGGAACCACCGATGGTCAGTGTGGATTCGAGCCTGATGGGCGAGCCGAACTTGACGACCGAGATGCTAAACGTATTCTCGCAGTGACCCAGAAGGGTGACGCATGGATTCGTGCGTTACAGGATACTATTCGTGAACTGCAACGTAAGCAGGAAATCAAGTAAGGAGGCAATGTGTCTACTCAATCCAATCGTAATGCGCTCGTAGTGGCGCAACTGAAAGGAGACTTCGTGGCGTTCCTATTCGTCTTATgg                                                                                                                     <  1:63449/301‑1 (MQ=255)`  
                                                                                                                                                                                         `atgtggataagagacagaTAAGTGGTTGCGCGTCAGAGTCAAAACTACCGGAACCTCCGATGGTCAGGGTGGATTCGAGCCTGATGGTCGAGCCGAACTTGACGACCGAGATGCTAAACGTATTCTCGCAGTGACCCAGAAGGGTGACGCATGGATTCGTGCGTTACAGGATACTATTCGTGAACTGCAACGTAAGTAGGAAATCAAGTAAGGAGGCAATGTGTCTACTCAATCCAATCGTAATACGCTCGTAGTGGCGCAACTGAAAGGAGACTTCGTGGCGTTCCTATTCGTCTTATgg                                                                                                                     <  1:182523/284‑1 (MQ=255)`  
                                                                                                                                                                                          `gATTTGCGTAGCGACAATAAGCGGTTGCGCGTCAGAGTCAAAACTACCGGAACCTCCGATGGTCAGTGTGGATTCGAGCCTGATGGTCTAGCCGAACTTGACGACCGAGATGCTAAACGTATTCTCGCAGTGACCCAGAAGGGTGACGCATGGATTCGTGCGTTACAGGATACTATTCGTGAACTGCAACGTAAGCAGGAAATCAAGTAAGGAGGCAATGTGTCTACTCAATCCAATCGTAATGCGCTCGTAGTGGCGCAACTGAAAGGAGACTTCCTgtctcttatacacatctccgagc                                                                                                                    >  1:1735/1‑279 (MQ=255)`  
                                                                                                                                                                                          `gATTTGCGTAGCGACAATAAGCGGTTGCGCGTCAGAGTCAAAACTACCGGAACCTCCGATGGTCAGTGTGGATTCGAGCCTGATGGTCGATCCGAACTTGACGACCGAGATGCTAAACGTATTCTCGCAGTGACCCAGAAGGGTGACGCATGGAATCGTGCGTTACAGGATACTATTAGTGAACTGCAACGTAAGTAGGAAATCAAGTAAGGAGGCAATGTGTCTACTCAATCCAATCGTAATGCGCTCGTAGTGGCGCAACTGTAAGAAGACTTCGTGGCGTTCCTATTCGGCTGATGGa                                                                                                                    >  1:519756/1‑301 (MQ=255)`  
                                                                                                                                                                                          `gATTTGCGTAGCGACAATAAGCGGTTGCGCGTCAGAGTCAAAACTACCGGAACCTCCGATGGTCAGTGTGGATTCGAGCCTGATGGTCGAGCCGAACTTGACGACCGAGATGCTAAACGTATTCTCGCAGTGACCCAGAAGGGTGACGCATGGATTCCTGCGTTACAGGATACTATTCGTTAACTGCAACGTAAGTAGGAAATCAAGTAAGGAGGCAATGTGTCTACTCAATCCAATCGTAATGCGCTCGTAGTGGCGCAACTGAAAGGAGACTTCGTGGCGTTCCTATTCGTCTTATGGa                                                                                                                    >  1:164752/1‑301 (MQ=255)`  
                                                                                                                                                                                                          `aTAAGCGGTTGCGCGTCAGAGTCAAAACTACCGGAACCTCCGATGGTCAGTGTGGATTCGAGCCTGATGGTCGAGCCGAACTTGACGACCGAGATGCTAAACGTATTCTCGCAGTGACCCAGAAGGGTGACGCATGGATTCGTGCGTTACAGGATACTATTCGTGAACTGCAACGTAAGCAGGAAATCAAGTAAGGAGGCAATGTGTCTACTCAATCCAATCGTAATGCGCTCGTAGTGGCGCAACTGAAAGGAGACTTCGTGGCGTTCCTATTCGTCTTATGGAAGGCGCTAAACCTAcc                                                                                                    <  1:8348/301‑1 (MQ=255)`  
                                                                                                                                                                                                            `aaGCGGTTGCTCGTCAGAGTCAAAACTACCGGAACCTCCGATGGTCAGTGTGGATTCGAGCCTGATGGTCGAGCCGAACTTGACGACCGAGATGCTAAACGTATTCTCGCAGTGACCCAGAAGGGTGACGCATGGATTCGTGCGTTACAGGATACTATTCGTGAACTGCAACGTAAGCAGGAAATCAAGTAAGGAGGCAATGTGTCTACTCAATCCAATCGTAATGCGCTCGTAGTGGCGCAACTGAAAGGAGACTTCGTGGCGTTCCTATTCGTCTTATGGAAGGCGCTAAACCTACCgg                                                                                                  <  1:525676/301‑1 (MQ=255)`  
                                                                                                                                                                                                            `aaGCGGTTGCGCGTCAGAGTCAAAACTACCGGAACCTCCGATGGTCAGTGTGGATTCGGGCCTGATGGTCGAGCCGAACTTGACGACCGAGATGCTAAACGTATTCTCGCAGTGACCCAGAAGGGTGACGCATGGATTCGTGCGTTACAGGATACTATTCGTGAACTGCAACGTAAGCAGGAAATCAAGTAAGGAGGCAATGTGTCTACTCAATCCAATCGTAATGCGCTCGTAGTGGCGCAACTGAAAGGAGACTTCGTGGCGTTCCTATTCGTCTTATGGAAGGCGCTAAACCTACCgg                                                                                                  <  1:339123/301‑1 (MQ=255)`  
                                                                                                                                                                                                                `ggTTGCGCGTCAGAGTCAAAACTACCGGAACCTCCGATGGTCAGTGTGGATTCGAGCCTGATGGTCGAGCCGAACTTGACGACCGAGATGCTAAACGTATTCTCGCAGTGACCCAGAAGGGTGACGCATGGATTCGTGCGTTACAGGATACTATTCGTGAACTGCAACGTAAGTAGGAAATCAAGTAAGGAGGCAATGTGTCTACTCAATCCAATCGTAATGCGCTCGTAGTGGCGCAACTGAAAGGAGACTTCGTGGCGTTCCTATTCGTCTTATGGAAGGCGCTAAACCTACCGGTGcc                                                                                              <  1:490110/301‑1 (MQ=255)`  
                                                                                                                                                                                                                       `ggTCAGAGTAAAAACTACCGGAACCTCCGATGGTCAGGGGGGATTCGAGCCTGATGGTCGAGCCGAACTTGACGACCGAGATGCTAAACGTATTCTCGCAGTGACCCAGAAGGGTGACGCATGGATTCGTGCGTTACAGGATACTATTCGTGAACTGCAACGTAAGTAGGAAATCAAGTAAGGAGGCAATGTGTCTACTCAATCCAATCGTAATGCGCTCGTAGTGGCGCAACTGAAAGGAGACTTCGTGGCGTTCCTATTCGTCTTATGGAAGGCGCTAAACCTACCGGTGCCCACTAAg                                                                                       <  1:226209/300‑1 (MQ=255)`  
                                                                                                                                                                                                                                    `gagatgtgtataagagacagTCAGTGTGGATTCGAGCCTGATGGGCGAGCCGAACTTGACGACCGAGATGCTAAACGTATTCTCGCAGTGACCCAGAAGGGTGACGCATGGATTCGTGCGTTACAGGATACTATTCGTGAACTGCAACGTAAGCAGGAAATCAAGTAAGGAGGCAATGTGTCTACTCAATCCAATCGTAATGCGCTCGTAGTGGCGCAACTGAAAGGAGACTTCGTGGCGTTCCTATTCGTCTTATGGAAGGCGCTAAACCTACCGGTGCCCACTAAGTGTCAGATTGACa                                                                          <  1:122331/282‑1 (MQ=255)`  
                                                                                                                                                                                                                                    `aaagagacagccTCCGATGGTCAGTGTGGATTCGAGCCTGATGGTCGAGCCGAAATTGACGACCGAGATGCTAAACGTATTCTCGCAGTGACCCAGAAGGGTGACGCATGGATTCGTGCGTTACAGGATACTATTCGTGAACTGCAACGTAAGCAGGAAATCAAGTAAGGAGGCAATGTGTCTACTCAATCCAATCGTAATGCGCTCGTAGTGGCGCAACTGAAAGGAGACTTCGTGGCGTTCCTATTCGTCTTATGGAAGGCGCTAAACCTACCGGTGCCCACTAAGTGTCAGATTGACa                                                                          <  1:136943/291‑1 (MQ=255)`  
                                                                                                                                                                                                                                         `gtataagagacagggTCAGTGTGGATTCGAGCCTGATGGTCGAGCCGAACTTGACGACCGAGATGCTAAACGTATTCTCGCAGTGACCCAGAAGGGTGACGCATGGATTCGTGCGTTACAGGATACTATTCGTGAACTGCAACGTAAGTAGGAAATCAAGTAAGGAGGCAATGTGTCTACTCAATCCAATCGTAATGCGCTCGTAGTGGCGCAACTGAAAGGAGACTTCGTGGCGTTCCTATTCGTCTTATGGAAGGCGCTAAACCTACCGGTGCCCACTAAGTGTCAGATTGACATGGCt                                                                     <  1:290870/288‑1 (MQ=255)`  
                                                                                                                                                                                                                                            `aaCCTCCGATGGTCAGTGTGGATTCGAGCCTGATGGTCGAGCCGAACTTGACGACCGAGATGCTAAACGTATTCTCGCAGTGACCCAGAAGGGTGACGCATGGATTCGTGCGTTACAGGATACTATTCGTGAACTGCAACGTAAGCAGGAAATCAAGTAAGGAGGCAATGTGTCTACTCAATCCAATCGTAATGCGCTCGTAGTGGCGCAACTGAAAGGAGACTTCGTGGCGTTCCTATTCGTCTTATGGAAGGCGCTAAACCTACCGGTGCCCACTAAGTGTCAGAtctgtctcttatac                                                                  >  1:467018/1‑288 (MQ=255)`  
                                                                                                                                                                                                                                             `accgtcGATGGTCAGTGTGGATTCGAGCCTGATGGTCGAGCCGAACTTGACGACCGAGATGCTAAACGTATTCTCGCAGTGACCCAGAAGGGTGACGCATGGATTCGTGCGTTACAGGATACTATTCGTGAACTGCAACGTAAGCAGGAAATCAAGTAAGGAGGCAATGTGTCTACTCAATCCAATCGTAATGCGCTCGTAGTGGCGCAACTGAAAGGAGACTTCGTGGCGTTCCTATTCGTCTTATGGAAGGCGCTAAACCTACCGGTGCCCACTAAGTGTCAGATTGACATGGCTAAgg                                                                 <  1:756128/296‑1 (MQ=255)`  
                                                                                                                                                                                                                                              `ccccccatgggaattagggATTCGAGCCTGATGGTCGAGCCGAACTTGACGACCGAGATGCTAAACGTATTCTCGCAGTGACCCAGAAGGGTGACGCATGGATTCGTGCGTTACAGGATACTATTCGTGAACTGCAACGTAAGCAGGAAATCAAGTAAGGGGGCAATGTGTCTACTCCATCCAATCGTGATGCGCTCGTAGTAGAGCAACTGAAAGGAGACTTCGTGGCGTTCCTATTCGTCTTATGGAAGGCGCTAAACCTACCGGTGCCCACTAAGTGTCAGATTGACATGGCTAAGGt                                                                <  1:449175/284‑1 (MQ=255)`  
                                                                                                                                                                                                                                              `ccTGCGATGGTCAGTGTGGATTCGAGGCTGATGGTCGAGCCGAACTTGACGACCGAGATGCTAAACGTATGCTCGCAGTGACCCAGAAGGGTGACGAATGGATTCGTGCGTTACATGATACTATTCGTGAACTGCAACGTAAGCAGGAAATCAAGTAAGGAGGCAATGTGTCTACTCAATCCAATCGTAATGCGCTCGTAGTGGCGCAACTGAAAGGAGACTTCGTGGCGTTCCTATTCGTCTTATGGAAGGCGCTAAACCTACCGGTGCCCACTAAGTGTCAGATTGACATGGCTAAGGt                                                                <  1:747842/301‑1 (MQ=255)`  
                                                                                                                                                                                                                                              `ccTCTGATGGTAAGTGTGGATTCGGGCCTGATGGTCGAGCCGAACTTGACGACCGAGATGCTAAACGTATTCTCGCAGTGACCCAGAAGGGTGACGCATGGATTCGTGCGTTACAGGATACTATTCGTGAACTGCAACGTAAGTAGGAAATCAAGTAAGGAGGCAATGTGTCTACTCAATCCAATCGTAATTCGCTCGTAGTGGCGCAACTGAAAGGAGACTTCGTGGCGTTCCTATTCGTCTTATGGAAGGCGCTAAACCTACCGGTGCCCACTAAGTGTCAGATTGACATGGCTAAGGt                                                                <  1:475132/301‑1 (MQ=255)`  
                                                                                                                                                                                                                                                     `tGGTCAGTGTGTATTCGAGCCAGATGTTCGAGCCGAACGTGACGACCGAGATGCTAAACGTATTCTCGCAGTGACCCAGAAGGGTGACGCATGGATTCGGTCGTTACAGGATACTATTCGTGAACTGCAACATAAGCAGTAAATCAAGTAAGGAGGCAATGTGTCTACTCAATCCAATCGTAATGCGCTCGTAGTGGCTCAACTGAAAGGAGACTTCGTGGCGTTCCTATTCGTCTTATGGAAGGCGCTAAACCTACCGGTGCCCACTAAGTGTCAGATTGACATGGCTAAGGTGCTGGCg                                                         <  1:113633/301‑1 (MQ=255)`  
                                                                                                                                                                                                                                                      `ggTCAGTGTGGATTCGAGCCTGATGGTCGAGCCGAACTTGACGACCGAGATGCTAAACGTATTCTCGCAGTGACCCAGAAGGGTGACGCATGGATTCGTGCGTTACAGGATACTATTCGTGAACTGCAACGTAAGTAGGAAATCAAGTAAGGAGGCAATGTGACTACTCAATCCAATCGTAATGCGCTCGTAGTGGCGCAACTGAAAGGAGACTTCGTGGCGTTCCTATTCGTCTTATGGAAGGCGCTAAACCTACCGGTGCCCACTAAGTGTCAGATTGACATGGCTAAGGTGCTGGCGa                                                        >  1:531369/1‑301 (MQ=255)`  
                                                                                                                                                                                                                                                           `gtgtGGATTCGAGCCTGATGGTCGAGCCGAACTTGACTACCGAGATGCTAAACGTATTCTCGCAGTGACCCAGAAGGGTGACGCATGGATTCGTGCGTTACAGGATACTATTCGTGAACTGCAACGTAAGTAGGAAATCAAGTAAGGAGGCAATGTGTCTACTCAATCCAATCGTAATGCGCTCGTAGTGGCGCAACTGAAAGGAGACTTCGTGGCGTTCCTATTCGTCTTATGGAAGGCGCTAAACCTACCGGTGCCCACTAAGTGTCAGATTGACATGGCTAAGGtctgtctcttataa                                                   >  1:391109/1‑288 (MQ=255)`  
                                                                                                                                                                                                                                                           `gtgtGGATTCGAGCCTGATGGTCGAGCCGAACTTGACGACCGAGATGCTAAACGTATTCTCGCAGTGACACAGAAGGGTGACGCATGGATTCGTGCGTTACAGGATACTATTCGTGAACTGCAACGTAAGCAGGAAATCAAGTAAGGAGGCAATGTGTCTACTCAATCCAATCGTAATGCGCTCGTAGTGGCGCAACTGAAAGGAGACTTCGTGGCGTTCCTATTCGTCTTATGGAAGGCGATAAACCTACCGGTGCCCACTAAGTGTCAGATTGACATGGCTAAGGtctgtctcttatac                                                   >  1:793091/1‑288 (MQ=255)`  
                                                                                                                                                                                                                                                                 `aTTCGAGCCTGATGGTCGAGCCGAACTTGACGACCGAGATGCTAAACGTATTCTCGCAGTGACCCAGAAGGGTGACGCATGGATTCGTGCGTTACAGGATACTTTTCGTGAACTGCAACGTAAGTAGGAAATCAAGTAAGGAGGCAATGTGTCTACTCAATCCAATCGTAATGCGCTCGTAGTGGCGCAACTGAAAGGAGACTTCGTGGCGTTCCTATTCGTCTTATGGAAGGCGCTAAACCTACCGGTGCCCACTAAGTGTCAGATTGACATGGCTAAGGTGCTGGCGAATGGAGacaac                                             <  1:317939/301‑1 (MQ=255)`  
                                                                                                                                                                                                                                                                 `aTTCGAGCCTGATGGTCGAGCCGAACTTGACGACCGAGATGCTAAACGTATTCTCGCAGTGACCCAGAAGGGTGACGCATGGATTCGTGCGTTACAGGATACTATTCGTGAACTGCAACGTAAGCAGGAAATCAAGTAAGGAGGCAATGTGTCTACTCAATCCAATCGTAATGCGCTCGTAGTGGCGCAACTGAAAGGAGACTTCGTGGCGTTCCTATTCGTCTTATGGAAGGCGCTAAACCTACCGGTGCCCACTAAGTGTCAGATTGACATGGCTAAGGTGCTGGCGAATGGAGacaac                                             <  1:39844/301‑1 (MQ=255)`  
                                                                                                                                                                                                                                                                 `aTTCGAGCCTGATGGTCGAGCCGAACTTGACGACCGAGATGCTAAACGTATTCACGCAGTGACCCAGAAGGGTGACGCATGGATTCGTGCGTTACAGGATACTATTCGTGAACTGCAACGTAAGCAGGAAATCAAGTAAGGAGGCAATGTGTCTACTCACTCCAATCGTAATGCGCTCGTAGTGGCGCAACTGAAAGGAGACTTCGTGGCGTTCCTATTCGTCTTATGGAAGGCGCTAAACCTACCGGTGCCCACTAAGTGTCAGATTGACATGGCTAAGGTGCTGGCGAATGGAGacaac                                             <  1:277026/301‑1 (MQ=255)`  
                                                                                                                                                                                                                                                                        `ccTGATGGTCGAGCCGAACTTGACGACCGAGATGCTAAACGTATTCTCGCAGTGACCCAGAAGGGTGACGCATGGATTCGTGCGTTACAGGATACTATTCGTGAACTGCAACGTAAGCAGGAAATCAAGTAAGGAGGCAATGTGTCTACTCAATCCAATCGTAATGCGCTCGTAGTGGCGCAACTGAAAGGAGACTTCGTGGCGTTCCTATTCGTCTTATGGAAGGCGCTAAACCTACCGGTGCCCACTAAGTGTCAGATTGACATGGCTAAGGTGCTGGCGAATGGAGACAACaagaagg                                      >  1:218619/1‑300 (MQ=255)`  
                                                                                                                                                                                                                                                                          `tGATGGTCGAGCCGAACTTGACGACCGAGATGCTAAACGTATTCTCGCAGTGACCCAGAAGGGTGACGCATGGATTCGTGCGTTACAGGATACTATTCGTGAACTGCAACGTAAGCAGGAAATCAAGTAAGGAGGCAATGTGTCTACTCAATCCAATCGTAATGCGCTCGTAGTGGCGCAACTGAAAGGAGACTTCGTGGCGTTCCTATTCGTCTTATGGAAGGCGCTAAACCTACCGGTGCCCACTAAGTGTCAGATTGACATGGCTAAGGTGCTGGCGAATGGAGACAACAAGAAGttt                                    >  1:587852/1‑300 (MQ=255)`  
                                                                                                                                                                                                                                                                          `tGATGGTCGAGCCGAACTTGACGACCGAGATGCTAAACGTATTCTCGCAGTGACCCAGAAGGGTGACGCATGGATTCGTGCGTTACAGGATACTATTCGTGAACTGCAACGTAAGCAGGAAATCAAGTAAGGAGGCAATGTGTCTACTCAATCCAATCGTAATGCGCTCGTAGCGGCGCAACTGAAAGGAGACTTCGTGGCGTTCCTATTCGTCTTATGGAAGGCGCTAAACCTACCGGTGCCCACTAAGTGTCAGATTGACATGGCTAAGGTGCTGGCGAATggctgtctcttatacaca                                    >  1:556946/1‑285 (MQ=255)`  
                                                                                                                                                                                                                                                                            `aTGGTCGAGCCGAACTTGACGACCGAGATGCTAAACGTATTCTCGCAGTGACCCAGAAGGGTGACGCATGGATTCGTGAGTTACAGGATACTATTCGTGAACTGCAACGTAAGAAGGAAATCAAGTAAGGAGGCAATGTGTCTACTCAATCCAATCGTAATGCGCTCGTAGTGGCGCAACTGAAAGGAGACTTCGTGGCGTTCCTATTCGTCTTATGGAAGGCGCTAAACCTACCGGTGCCCACTAAGTGTCAGATTGACATGGCTAAGGTGCTGGCGAATGGAGACAACAAGAAGTTCAt                                  <  1:814493/301‑1 (MQ=255)`  
                                                                                                                                                                                                                                                                             `tGGTCGAGCCGAACTTGACGACCGAGATGCTAAACGTATTCTCGCAGTGACCCAGAAGGGTGACGCATGGATTCGTGCGTTACAGGATACTATTCGTGAACTGCAACGTAAGTAGGAAATCAAGTAAGGAGGCAATGTGTCTACTCAATCCAATCGTAATGCGCTCGTAGTGGCGCAACTGAAAGGAGACTTCGTGGCGTTCCTATTCGTCTTATGGAAGGCGCTAAACCTACCGGTGCCCACTAAGTGTCAGATTGACATGGCTAAGGTGCTGGCGAATGGAGACAACAAGAAGTTCATc                                 <  1:100017/301‑1 (MQ=255)`  
                                                                                                                                                                                                                                                                               `ggcGAGCCGAACTTGACGACCGGGATGGTAAACGTATTCTCGCAGTGACCCAGAAGGGTGACGCATGGATTCGTGCGTTACAGGAGAATATTCGTGAACTGCAACGTAAGCAGGAAAGCAAGTAAGGAGGCAATGTGTCTACTCACTCCCAGCGTAATGCGCTCGTAGTGGCGCAACTGAAAGGAGACTTCGTGGCGTTCCTATTCGTCTTATGGAAGGCGCTAAACCTACCGGTGCCCACTAAGTGTCAGATTGACATGGCTAAGGTGCTGGCGAATGGAGACAACAAGAAGTTCATCtt                               <  1:715055/299‑1 (MQ=255)`  
                                                                                                                                                                                                                                                                                        `aaCTTGACGACCGAGATGCTAAACGTATTCTCGCAGTGACCCAGAAGGGTGACGCATGGATTCGTGCGTTACAGGATACTATTCGTGAACTGCAACGTAAGTAGGAAATCAAGTAAGGAGGCAATGTGTCTACTCAATCCAATCGTAATGCGCTCGTAGTGGCGCAACTGAAAGGAGACTTCGTGGCGTTCCTATTCGTCTTATGGAAGGCGCTAAACCTACCGGTGCCCACTAAGTGTCAGATTGACATGGCTAAGGTGCTGGCGAATGGAGACAACAAGAAGTTCATCTTACAGGCttt                      >  1:683833/1‑301 (MQ=255)`  
                                                                                                                                                                                                                                                                                        `aaCTTGACGACCGAGATGCTAAACGTATTCTCGCAGTGACCCAGAAGGGTGACGCATGGATTCGTGCGTTACAGGATACTATTCGTGAACTGCAACGTAAGCAGGAAATCAAGTAAGGAGGCAATGTGTCTACTCAATCCAATCGTAATGCGCTCGTAGTGGCGCAACTGAAAGGAGACTTCGTGGCGTTCCTATTCGTCTTATGGAAGGCGCTAAACCTACCGGTGCCCACTAAGTGTCAGATTGACATGGCTAAGGTGCTGGCGAATggctgtctcttatacacatctccgagcccacg                      >  1:256426/1‑271 (MQ=255)`  
                                                                                                                                                                                                                                                                                          `cTTGACGACCGAGATGCTAAACGTATTCTCGCAGTGACCCAGAAGGGTGACGCATGGATTCGTGCGTTACAGGATACTATTCGTGAACTGCAACGTAAGTAGGAAATCAAGTAAGAAGGCAATGTGTCTACTCAATCCAATCGTAATGCGCTCGTAGTGGCGCAACTGAAAGGAGACTTCGTGGCGTTCCTATTCGTCTTATGGAAGGCGCTAAACCTACCGGTGCCCACTAAGTGTCAGATTGACATGGCTAAGGTGCTGGCGAATGGAGACAACAAGAAGTTCATCTTACAGGCTTTcc                    >  1:442244/1‑301 (MQ=255)`  
                                                                                                                                                                                                                                                                                          `cTTGACGACCGAGATGCTAAACGTATTCTCGCAGTGACCCAGAAGGGTGACGCATGGATTCGTGCGTTACAGGATACTATTCGTGAACTGCAACGTAAGCAGGAAATCAAGTAAGGAGGCAATGTGTCTACTCAATCCAATCGTAATGCGCTCGTAGTGGCGCAACTGAAAGGAGACTTCGTGGCGTTCCTATTCGTCTTATGGAAGGCGCTAAACCTACCGGTGCCCACTAAGTGTCAGATTGACATGGCTAAGGTGCTGGCGAATGGAGACAACAAGAAGTTCATCTTACAGGCTTTcc                    >  1:643022/1‑301 (MQ=255)`  
                                                                                                                                                                                                                                                                                            `tGACGACCGAGATGCTAAACGTATTCTCGCAGTGACCCAGAAGGGTGACGCATGGATTCGTGCGTTACAGGATACTATTCGTGAACTGCAACGTAAGCAGGAAATCAAGTAAGGAGGCAATGTGTCTACTCAATCCAATCGTAATGCGCTCGTAGTGGCGCAACTGAAAGGAGACTTCGTGGCGTTCCTATTCGTCTTATGGAAGGCGCTAAACCTACCGGTGCCCACTAAGTGTCAGATTGACATGGCTAAGGTGCTGGCGAATGGAGACAACAAGAAGTTCATCTTACAGGCTTTCCGt                  >  1:428844/1‑301 (MQ=255)`  
                                                                                                                                                                                                                                                                                                      `gaTGCTAAACGTATTCTCGCAGTGACCCAGAAGGGTGACGAATGGATTCGTGCGTTACAGGATACTATTCGTGAACTGCAACGTAAGCAGGAAATCAAGTAAGGAGGCAATGTGTCTACTCAATCCAATCGTAATGCGCTCGTAGTGGCGCAAATGAAAGGAGACTTCGTGGCGTTCCTATACGTCTTATGGAAGGCGCTAAACCTACCGGTGCCCACTAAGTGTCAGATTGACATGGCTAAGGTGCTGGCGAATGGAGACAACAAGAAGTTCATCTTACAGGCTTTCCGTGGTATCGGTa        <  1:549130/301‑1 (MQ=255)`  
                                                                                                                                                                                                                                                                                                        `tGCTAAACGTATTCTCGCAGTGACCCAGAAGGGTGACGCATGGATTCGTGCGTTACAGGATACTATTCGTGAACTGCAACGTAAGTAGGAAATCAAGTAAGGAGGCAATATGTCTACTCAATCCAATCGTAATGCGCTCGTAGTGGCGCAACTGAAAGGAGACTTCGTGGCGTTCCTATTCGTCTTATGGAAGGCGCTAAACCTACCGGTGCCCACTAAGTGTCAGATTGACATGGCTAAGGTGCTGGCGAATGGAGACAACAAGAAGTTCATCTTACAGGCTTTCCGTGGTATCGGTAAg      >  1:626337/1‑301 (MQ=255)`  
                                                                                                                                                                                                                                                                                                         `gCTAAACGTATTCTCGCAGTGACCCAGAAGGGTGACGCATGGATTCGTGCGTTACAGGATACTATTCGTGAACTGCAACGTAAGCAGGAAATCAAGTAAGGAGGCAATGTGTCTACTCAATCCAATCGTAATGCGCTCGTAGTGGCGCAACTGAAAGGAGACTTCGTGGCGTTCCTATTCGTCTTATGGAAGGCGCTAAACCTACCGGTGCCCACTAAGTGTCAGATTGACATGGCTAAGGTGCTGGCGAATGGAGACAACAAGAAGTTCATCTTACAGGCTTTCCGTGGTATCGGTactg     >  1:265798/1‑298 (MQ=255)`  

                                                                                                                                                                                                                                                                                                            `|`                                                                                                                                                                                                                                                                                                              
`CTATTCGGGTTAGGATGGCATCTAGGGTCAGACTCAATGGACGCTAAATGGAAACAGGAGGTACACAATGAGTACGTTAAGAGAGTTGAGGCTGCGAAGAGCACTCAAAGAGCAATCGATGCGGTATCTGCTAAGTATCAAGAAGACCTTGCCGCGCTGGAAGGGAGCACTGATAGGATTATTTCTGATTTGCGTAGCGACAATAAGCGGTTGCGCGTCAGAGTCAAAACTACCGGAACCTCCGATGGTCAGTGTGGATTCGAGCCTGATGGTCGAGCCGAACTTGACGACCGAGATGCTAAACGTATTCTCGCAGTGACCCAGAAGGGTGACGCATGGATTCGTGCGTTACAGGATACTATTCGTGAACTGCAACGTAAGTAGGAAATCAAGTAAGGAGGCAATGTGTCTACTCAATCCAATCGTAATGCGCTCGTAGTGGCGCAACTGAAAGGAGACTTCGTGGCGTTCCTATTCGTCTTATGGAAGGCGCTAAACCTACCGGTGCCCACTAAGTGTCAGATTGACATGGCTAAGGTGCTGGCGAATGGAGACAACAAGAAGTTCATCTTACAGGCTTTCCGTGGTATCGGTAAGTCGT  >  NC_001604.1/36965‑37565`

  

Alignment Legend

Aligned base mismatch/match (shaded by quality score): `ATCG/ATCG` `< 3 ≤` `ATCG/ATCG` `< 11 ≤` `ATCG/ATCG` `< 25 ≤` `ATCG/ATCG` `< 36 ≤` `ATCG/ATCG` `< 38 ≤` `ATCG/ATCG`

Unaligned base: `atcg`    Masked matching base: `atcg`    Alignment gap: `‑`    Deleted base: `‑`

Reads not counted as support for junction

read\_name Not counted due to insufficient overlap past the breakpoint.

read\_name Not counted due to not crossing MOB target site duplication.
